# Reconfiguring retention: a qualitative exploration of the lived experiences of female nurses in an arid rural setting in Turkana County, Kenya

**DOI:** 10.1080/16549716.2026.2675808

**Published:** 2026-05-21

**Authors:** Sif Sofie Vange, Mathilde Vraa-Jensen, Emma Borre Andersen, Lasse X. Jensen, Micah Matiang’i, Hesborn Wao, Siv Steffen Nygaard, Jane Brandt Sørensen

**Affiliations:** a Global Health Section, Department of Public Health, University of Copenhagen, Copenhagen, Denmark; b School of Medical Sciences, Amref International University, Nairobi, Kenya; c Individual Capacity Strengthening, African Population and Health Research Center, Nairobi, Kenya; d Social & Humanitarian, Novo Nordisk Foundation, Copenhagen, Denmark

**Keywords:** Ethnography, nursing, outmigration, workforce mobility, East Africa

## Abstract

**Background:**

Retention is especially critical in rural and underserved areas of East Africa, such as Turkana County in Kenya, where resource limitations, harsh conditions, and a lack of professional development opportunities deter nurses from staying. In addition, female nurses face unique challenges shaped by systemic inequities and gendered expectations, influencing their retention.

**Objective:**

The aim of this study was to explore factors influencing retention of female nurses in Turkana and to offer new perspectives on the concept of retention from underserved regions in East Africa.

**Methods:**

This quasi-ethnographic study was conducted in an arid rural setting in Turkana, in 2024. Data were collected through semi-structured interviews with 21 female nurses and 8 local health administrators and through participant observations at 4 health facilities. We used thematic network analysis guided by an abductive approach.

**Results:**

Female nurses in Turkana navigate a paradox of staff shortages alongside high unemployment, leaving many feeling stuck in occupational limbo or permanent liminality, hoping, and working toward better opportunities for themselves and their families. Although the motivation to work as nurses persists, challenging working conditions lead many to aspire to migrate abroad.

**Conclusion:**

This study demonstrates the complex interplay of local and global dynamics driving retention of female nurses in Turkana. Perceived workforce stability is largely due to immobility caused by limited alternatives and systemic constraints. This immobility masks dissatisfaction, making the healthcare system in Turkana vulnerable to outmigration. Findings highlight the need for holistic, gender-sensitive policies that enhance rural career pathways for female nurses.

## KEY POINTS

- **Main findings**: Retention of nurses is shaped by both local structural constraints and broader global dynamics, resulting in a sense of being stuck in positions, while simultaneously channelling hopes and efforts toward mobility abroad.
- **Added knowledge**: Retention of nurses should be understood by considering both local and global dynamics and moving beyond numbers in a system to capture the nuanced realities of retention, including the co-existence of immobility and dissatisfaction.
- **Global health impact for policy and action**: Future strategies to strengthen retention in arid and rural settings should aim to reduce inequities in career advancement, consider the influence of global migration on local retention, and understand its impact on already strained health services.

## Background

Effective healthcare workforce retention is essential to mitigate the rising global shortage of healthcare professionals, which is projected to reach 11 million by 2030 [1]. These shortages weaken healthcare service delivery and disproportionately affect vulnerable populations [2]. Under-investment in the health sector, coupled with a mismatch between health system needs and workforce capacity, exacerbates this crisis [1], especially in low- and middle-income countries (LMICs) [3]. Sub-Saharan Africa (SSA) is particularly affected as it bears 25% of the world’s disease burden yet has only 4% of the world’s healthcare workforce [4]. Health worker shortages in arid rural areas of East Africa are shaped by a complex interplay between personal and professional incentives and circumstances such as salary, promotion prospects, and community and family ties [5–11]. In Kenya, there are disparities in health workforce capacity between its 47 counties, with rural areas such as Turkana County facing one of the nation’s lowest nurse-to-population ratios [12,13]. Qualitative research from Kenya shows how staffing shortages, combined with high patient loads and limited resources, force nurses to prioritise specific tasks, affecting both care quality and job satisfaction [14].

Health workforce retention refers to the ability of healthcare systems or organizations to maintain their workforce by minimising staff turnover and ensuring that professionals remain in their positions [15]. Retention is critical in rural areas of SSA, where limited resources, challenging environmental conditions, and scarce opportunities for professional development discourage nurses from staying in underserved areas [6,9,16]. Nurses are the largest group of health professionals in rural settings, and they play a central role in sustaining rural healthcare systems [17–19]. It is, therefore, essential to understand their experiences in order to improve health workforce retention. Focused research can yield critical insights into workforce shortages and maldistribution and inform policies fostering supportive work environments.

Gender likewise plays a critical role in shaping the experiences and career trajectories of healthcare workers, especially in rural and harsh environments where systemic inequities intersect with societal expectations and create unique challenges for female professionals [10,20–23]. This is evident in the discrimination faced by women in healthcare, as motherhood and family responsibilities often lead them to be overlooked for leadership roles [24]. A recent study found that policies in Kenya supporting mentorship programs, paid leave, lactation breaks, and childcare services are crucial for retaining nurses – particularly women – in the health workforce. However, these policies are inconsistently implemented across facilities [24]. This highlights the intricacies of healthcare worker retention in underserved areas, emphasising the need for in-depth, context-specific research.

Previous research highlights a significant gap in understanding female nurses’ specific experiences in rural areas [10,21]. Thus, the aim of this study is to explore factors influencing retention of female nurses in Turkana County, and to offer new perspectives from underserved regions in East Africa to inform future research and policy.

## Methods

### Positionality

Research was carried out by an international and interdisciplinary research team from Kenya and Denmark with backgrounds in anthropology, global health, nursing, educational research, and public health. Planning and data analysis were collaborative, and data collection was conducted by three Danish women (SSV, MVJ, EBA) with bachelor’s degrees in Anthropology who were completing their master’s degrees in Global Health. During data collection, different personal characteristics guided their engagement with the participants, research team, and study topics. Unfamiliarity with the local language and physical differences often positioned them as outsiders – enabling them to question assumptions and, at times, access personal information. However, this unfamiliarity also posed challenges with interpreting contextual cues, which the researchers addressed through informal conversations with participants and local collaborators. Nevertheless, outsider positionality was not definite; shared gender and relational dynamics fostered a sense of trust and mutual understanding in conversations with female nurses, including topics such as children and breastfeeding. The positionality and diverse pre-understandings of all researchers involved shaped how the field was interpreted and represented and resulted in a collaboratively constructed analysis. We highlight this to emphasise that knowledge production is inherently relational and cannot be disentangled from interactions, contexts, and individuals involved in its creation.

### Study setting and data collection

The study was conducted in Turkana County, one of Kenya’s largest yet most marginalised counties [25]. Spanning 77,000 km2, Turkana is predominantly rural and characterised by arid and semi-arid landscapes. Socioeconomic vulnerability is high, with more than 80% of residents living below the national poverty line [25].

Data collection took place between August and September 2024 at four selected healthcare facilities across three sub-counties in Turkana. One level four facility served as our main field site, where nine visits allowed for greater familiarisation with the facility and staff. The three remaining facilities, including two level three health centres and one level four county hospitals, were visited to get a broader understanding of the multiple realities across health facilities in the county.

Nurses and local health administrators were recruited through non-random purposive sampling with the assistance of gatekeepers. Prior to arrival, we obtained permission from relevant authorities to collect data. Health facility management then conveyed the information about the project to the nurses. Female registered nurses employed in a temporary or permanent position were recruited as participants on a voluntary basis.

We employed a quasi-ethnographic study design where semi-structured interviews were carried out with female nurses and local health administrators, and observations were conducted at all facilities. Participant observations were applied to explore nurses’ daily routines and interactions, including with colleagues and patients, and to capture the interplay between organisational structures and informal workplace practices.

Semi-structured interviews followed interview guides that were refined and adapted throughout data collection. Thematic saturation was reached after approximately 15–18 interviews, at which point repeated patterns in the data continued to emerge. Interviews lasted between 21 and 106 minutes and were held in various locations, often wherever space was available. This included empty offices, staff areas within the facilities, outdoor facility grounds, and occasionally within or near wards, allowing nurses to return to their duties in due course. Consequently, bypassers occasionally compromised the level of privacy, so if patients entered the room during an interview, we paused the recording to ensure privacy and prioritise patient care. These situations may have influenced the depth and candour of the conversation. For health administrators, interviews were typically conducted in their offices. To acknowledge time and effort, we provided tokens of 1,000 Kenyan Shillings (~$7.74 USD) after interviews, as recommended by the Kenyan co-authors of this study. This modest, context-appropriate token that acknowledge participants’ contributions without creating undue influence aligns with guidance on participant compensation in low-resource settings [26]. Data collection was conducted in English without a translator due to the high level of English proficiency among nurses.

Participant observations were conducted at each facility, focusing on the nurses’ daily routines through their perspectives. This excludes private patient consultations. Observations and reflections were documented in field notes, written both during and after data collection to ensure that details were captured while still fresh in mind. The interviews were recorded and transcribed verbatim.

### Data analysis

We adopted an abductive approach, which emphasises the dynamic interplay between empirical data and theoretical constructs [27,28]. Our analytical approach, preliminary themes, and coding framework were refined and developed through familiarisation with the interviews and observation data and subsequent discussions within the wider research group.

Interview coding was first conducted independently by SSV, MVJ, and EBA, followed by a consensus-coding process to resolve discrepancies. All data was managed and analysed on NVivo. Codes were developed through thematic network analysis to ensure a structured categorical indexing of material. Basic themes were identified and iteratively refined as both the analysis and theoretical understanding evolved [28]. The emerging themes reflected key areas such as work environment, gender dynamics, and future aspirations, with a conceptual anchoring in our theoretical framework. Observation data and fieldnotes added valuable context to the findings. The interplay between the diverse background of the wider research group, reflexive field notes, participant observation data, and interview data ensured that the analysis was both grounded and contextually rich.

### Ethical considerations

The study received relevant ethical approval (see section on ethics and consent for further details) and was conducted per the Declaration of Helsinki [29] and the American Anthropological Association’s Seven Principles of Professional Responsibility on Ethics [30]. All participants provided informed, written consent. Data were pseudonymised by authors for both analysis and reporting. The presented findings are a synthesis of multiple nurses’ perspectives; identifying elements were removed or blended to maintain confidentiality while still preserving the depth of insight and conveying collective group experiences.

### Theoretical framework

This study draws on the concepts of *occupational limbo* and *permanent liminality* [31] to explore how female nurses experience their work lives. Occupational limbo is defined as a static, constrained state in which individuals feel stuck in undesirable roles with little hope for change, whereas permanent liminality describes a prolonged and ambiguous state of being in-between, marked by hope and active navigation and adaptation toward a better future [31]. To further understand its role in these states, we apply Alacovska’s conceptualisation of hope [32], which defines it as practical, daily actions and forward-looking thinking aimed at pursuing future possibilities. Hope, therefore, involves the belief that one’s actions can shape the future, fostering determination to move forward [32]. Specifically, we examine how different forms of hope emerge in these liminal spaces depending on the type of employment and career stage, enabling some nurses to envision and pursue change.

## Findings

### Participants characteristics

The study participants included female nurses and health administrators. We interviewed 21 female nurses working in level three and four healthcare facilities in Turkana County, except one who was employed at a level two facility. Lower-level facilities and male nurses were excluded to maintain a feasible research scope and focus on female nurses’ unique experience. To complement the nurses’ perspectives, we interviewed eight local health administrators involved in nursing education and employment in Turkana County. Table 1 details participant characteristics.

**Table 1.** Participant characteristics.

Characteristic		Nurses (*n* = 21)	Health administrators (*n* = 8)
Age	Average age	37 years	39 years
**Origin**	Turkana origin	9	5
	Non-Turkana origin	12	3
**Civil status**	Married	15	–
	Not married	6	–
**Children**	Has children	19	–
	No children	3	–
**Educational level**	Registered nurse	18	3
	Other degree in nursing	3	–
	Master’s degree	–	3
	Other	–	2

### Navigating systemic challenges

The nurses shared experiences of motivation and demotivation rooted in both the nature of the job and its systemic context. They described a passion for the nursing profession and helping the less fortunate, while also identifying sources of frustration. First, the increasing influence of personal connections in the allocation of professional benefits makes career advancement dependent not only on merit but also on ‘whom you know’. Second, a substantial sense of waiting was described regarding both their current and future jobs as nurses.

There are so many challenges that are beyond us. We are just there waiting … we are giving them [Turkana Government] time to do it. To do away with the challenges they are facing, so that they can meet our needs. – Akope, nurse

Akope’s words illustrate how waiting has become embedded in daily working life, signaling a broader experience of uncertainty rather than temporary delay. This waiting state reflects the systemic challenges straining health facilities, including resource shortages, incomplete infrastructure, and funding delays. Amid these inefficiencies, nurses in Turkana face uncertainty, and must navigate the paradox of simultaneous staff shortages and high unemployment rates. While an increasing number of qualified nurses are receiving training, many remain unemployed and unable to secure permanent positions despite facility needs. Some aimed to advance through further training or education, while others wished to leave for better-equipped facilities or out-migrate from Turkana to counties or countries with more opportunity. This staffing challenge is exacerbated by the presence of ‘ghost workers’, described by nurses and health administrators as individuals who remain on the payroll without currently working, creating the illusion of a better-staffed healthcare system. Some may have retired, resigned, or even passed away, yet remain listed as active employees due to systemic negligence – or, as some suggest, deliberate misconduct by officials aiming to inflate the number of nurses on record. Many nurses described a similar issue without using the emic term ‘ghost workers’, where staff face an increased workload due to discrepancies between the number of recorded versus personnel actually present at the facility. Here, the healthcare system itself appears in an unresolved state, struggling to address both the visible and invisible gaps in staffing.

While most described frustration and stagnation, a few nurses expressed a commitment to staying in Turkana because they felt needed by the community. These perspectives show that working as a nurse in Turkana can also be tied to purpose and belonging.

### Stuck in the system

Before the 2010 constitution, nurses could request transfers between counties more easily, whereas transfers today have become increasingly challenging. The 2010 constitution, also referred to as devolution, decentralised the healthcare system to the county level. This leaves many nurses feeling stuck or confined to their current positions. Abigail, who has tried to relocate to her home county for years, describes the difficulties of transferring to a facility out of Turkana: ‘Since [the] devolution came there is no transfer. You have to swap`. Now, according to Abigail, nurses who wish to move counties must find someone in a similar position willing to swap places. At one point, Abigail identified a nurse from her home county who was willing to move to Turkana, but she changed her mind at the last minute, leaving Abigail unable to transfer. When discussing this matter, Abigail shrugged her shoulders and shook her head, signalling a quiet resignation. Abigail now awaits retirement as the only realistic means of returning home. Abigail’s feeling of confinement to Turkana reflects the notion of occupational limbo, where she has little hope for transferring to her home county.

A similar sense of confinement is expressed by Catherine, a nurse in her early thirties living alone in Turkana while her young child stays with her parents in another county. She expresses a strong desire to transfer closer to her child, but explains that her current situation in Turkana makes it difficult to swap:

Maybe since there is no one who is willing to come and work here, so since we have found ourselves here, we must just be here until somebody else is maybe willing to come. – Catherine, nurse

Cathrine’s words highlight how hope is contingent on circumstances beyond her control, reflecting a sense of restricted mobility. Her experience illustrates how after securing a job in Turkana, the current transfer system restricts nurses’ ability to move to other counties. Moreover, conversations with both health administrators and nurses revealed that high levels of unemployment further discourage nurses from leaving their positions.

The devolution, coupled with high unemployment, has limited job mobility for nurses like Abigail and Catherine, leaving them feeling stuck in their current positions and without hope of change. Their inability to envision a future in which they can choose their workplace can be understood as a form of occupational limbo. For them, waiting becomes a static and disheartening state, where the absence of hope of securing a transfer diminishes their motivation to pursue one. This reveals how waiting may become immobilising rather than motivating and aligns with the definition of occupational limbo.

### Locum nurses in permanent liminality

While Catherine and Abigail find themselves in a state of occupational limbo, other nurses navigate what can be described as permanent liminality. For them, hope becomes a driving force, fueling their ability to act, adapt, and persevere despite the uncertainties and challenges of their position. In these cases, waiting is active and future-oriented rather than passive.

In the Turkana County healthcare system, *locum contracts* are a common form of temporary employment that offer less security and benefits than permanent contracts. These 25-hour contracts play a vital role in maintaining service delivery by providing nurses with an entry point into permanent employment while offering flexibility to address chronic staffing shortages. In practice, however, locum positions frequently extend for years without resolution, leaving nurses continuously awaiting permanent employment.

Maraka and Karen are both employed on temporary locum contracts. For Maraka, the position as a locum nurse symbolises an endless waiting state. She describes her experience during one of our talks at the facility: ‘That is what we are waiting for. You are being told this month, next month, since last year. No feedback’. Her words reflect the uncertainty experienced by many locum nurses, where waiting becomes repetitive and lacks a clear endpoint. Karen recounts how her initial three-year locum contract was supposed to transition into permanent employment, yet remains unresolved: ‘I don’t know what will happen, because initially, the plan was that we do the [locum] contract for three years, then they will absorb us for permanent employment but it didn’t happen’.

These nurses occupy what Bamber and colleagues [31] describe as a state of permanent liminality. They endure prolonged uncertainty, awaiting permanent employment that rarely materialises. As such, locum nurses, like Maraka and Karen, are positioned as neither fully employed nor entirely unemployed. However, this permanent liminality also creates room for adaptability. Maraka, for example, volunteers at the facility beyond her contracted hours without pay, in hopes of increasing her chances of securing a permanent position. Other nurses explained that they leverage their locum position to work across multiple departments, thereby diversifying their skills and increasing their visibility. One locum nurse secured the opportunity to step in as a substitute in-charge during weekends or holidays when senior staff were off duty.

These actions exemplify how, in light of permanent liminality, locum nurses still take initiative, framing their unpaid efforts as investments in their potential future permanent employment opportunities. These efforts are not merely acts of submission to the system, but strategic moves within the constraints of their liminal positions to turn temporary work into permanent employment. For many locum nurses, hope serves as a motivator, encouraging them to navigate structural limitations creatively and to make their temporary roles meaningful. Simultaneously, nurses on locum contracts highlight the systemic pressures that normalise underpaid or unpaid labour within the healthcare sector. Since, healthcare facilities and county governments benefit from flexibility and cost-effectiveness of these initial short- term contracts, it is important to consider whether the long-term arrangements evolving from these contracts end up blending exploitation with hope and continue prioritizing the system over the individual.

### Aspirations for further training and outmigration

The nurses’ aspirations involved short- and long-term hopes shaped by a deep sense of uncertainty. This is not only confined to their immediate roles as nurses or locum nurses, but extends to broader visions of their futures, where they attempt to move forward within the constraints of an unpredictable job market and systemic injustices. These efforts reflect the tension between the search for better opportunities and the uncertainty of achieving them. These aspirations show how permanent liminality and hope extend beyond the current workplace and ties to imagined futures.

For several nurses, education and training opportunities were perceived as a stepping-stone to new positions in more specialised departments or even abroad. Valery, a young nurse with five years of experience in the field, shared her thoughts on accessing additional education:

Right now, I enjoy working at the intensive care unit and I don’t like, I don’t wish to be moved. So, my hope is that maybe in the future I can specialise in intensive care. – Valery, nurse

Valery has experienced being reassigned to various facility wards both on short notice and without choice, oftentimes merely discovering her name on a list at the administration ward or receiving a WhatsApp notification a few days before. Now, she hopes to stay at her current department by planning for specialisation, since specialised nurses have a lower chance of reassignment. This positions Valery in a state of permanent liminality – caught between the dread of anticipating potential reassignment and the prolonged planning and waiting for further education, which she sees as her pathway to professional stability. Her hopes for additional training are therefore not only about gaining new knowledge but also about reducing the uncertainty that defines her current position. Her account illustrates how hope may provide direction in the absence of secure or predictable opportunities.

The aspiration for further education extends beyond local career stability, representing a potential pathway to opportunities abroad. This was a prominent topic in conversations with the nurses; many found themselves in positions with limited resources and minimal possibilities for career advancement, dreaming of and waiting for a chance to work overseas.

Daisy and Akope shared this aspiration, both envisioning life abroad as a path to a brighter future for themselves and their families. Akope stated, ‘It’s not like I want to abandon my people or, but it’s exposure, greener pastures, new opportunities’. Similarly, Daisy imagined going ‘where the grass is greener’ without a particular country in mind. This shared metaphor of ‘greener pastures’ reflects the overarching hope for improved conditions rather than the commitment to a specific destination. For Daisy, Akope, and other nurses in Turkana, life abroad is idealised, and intertwined with notions of financial security and professional growth. A nurse named Grace expanded upon this aspiration of financial security:

I just feel like, you know, when you are dreaming, sometimes you want to get some vehicle money, like, you would prefer working where you would get more paid. So, you would wish at least one day, you go and work as a nurse in the U.S. and get some money to solve some problems …. – Grace, nurse

For Grace, migration to the U.S. represents a practical means of earning and accumulating wealth to overcome personal and financial challenges in her life. By framing migration as a tool for ‘solving some problems’, she underscores its functional value in improving her circumstances, making it an attractive and purposeful career decision.

Together, these narratives about improved compensation and working conditions abroad amplify the appeal of outmigration. The nurses’ current employment in Turkana – characterised by systemic challenges and limited opportunities – starkly contrasts their envisioned futures overseas, fueling their prolonged state of hope for change.

At the same time, many nurses acknowledge the significant challenges involved in leaving Kenya. The process is often long, expensive, and demanding; it involves completing required levels of training, submitting applications, attending interviews, and securing the necessary finances. These barriers lead to prolonged waiting periods, which in some cases become indefinite. For some of the female nurses, this process is further constrained by gendered familial obligations. Daisy pointed to the constraints of motherhood, noting:

… a thing that was limiting me are my kids. I was thinking that they are still young and, like, in some countries you have to go first and maybe settle and then take the kids later. … The young one is still breastfeeding. – Daisy, nurse

Her account of how caregiving responsibilities can delay women’s migration plans reflects the broader notion of the gendered division of family responsibilities. Thus, for many of the female nurses in Turkana, the vision of migration represents more than a career move; it represents a profound hope for greener pastures, labour mobility and a brighter future for them and their families.

## Discussion

This qualitative study shows that female nurse retention in Turkana is shaped by local structures and global migration dynamics, masking underlying dissatisfaction and straining the health system. The study explored factors influencing the retention of female nurses in Turkana County and offers new perspectives on the concept of retention in underserved regions in East Africa. Existing research on this topic often focuses on dissatisfaction [6,9,33], however a key contribution of this study is that apparent workforce stability can mask limited mobility, and does not always reflect satisfaction or strong workplace commitment. Where prior literature on retention often links workforce stability to workplace satisfaction, incentives, and supportive work environments [21,24,34], our findings indicate that in Turkana, retention also reflects a form of retention-by-default shaped by immobility and constrained choice. This insight adds nuance to conceptualisations of retention in research and highlights the need to reassess what long-term workforce stability means in underserved settings.

Our findings align with existing literature on retention from rural Uganda and Tanzania [5,9,11], emphasising how female nurses in Turkana are dissatisfied with the limited access to training, gendered barriers to career progression, inadequate infrastructure, and challenging working conditions. Previous research on women working in healthcare highlights how cultural norms systematically constrain their career progression. Caregiving remains undervalued, and male health workers are still prioritised for leadership positions despite policy promoting gender equity [35–37]. Our findings expand upon this, revealing how family obligations, shape female nurses’ mobility experiences as they postpone aspirations for outmigration until caregiving responsibilities are reduced. Research from other underserved settings, including Zimbabwe and Kenya, emphasises that retention improves when supportive environments are created through fair pay, manageable workloads, and opportunities for professional growth, such as training and career advancement [21,24,34]. In Turkana, our findings suggest that dissatisfaction is not just tied to the lack of supportive environments, but also to the disproportionate influence of personal relationships on professional opportunities.

Paradoxically, the lack of opportunity for professional mobility is also a key reason why many nurses remain in their current positions. Our study indicates that structural barriers make job transitions challenging, and individuals feel compelled to stay in their current roles despite dissatisfaction. In this context, immobility contributes to a skewed perception of retention, where stability in numbers does not necessarily reflect current workplace satisfaction, but rather a lack of alternatives. Thus, immobility and dissatisfaction coexist, both influencing the way retention manifests in Turkana.

Despite staying in place, many female nurses in our study – particularly those pursuing further education or considering outmigration – actively position themselves to leave when opportunities arise. For instance, nurses in our study described how those who obtain advanced training may not return to their previous facilities but instead seek other opportunities within or outside the county, or even abroad. Similar trends have been observed in rural Tanzania and Uganda, where nurses state that obtaining additional qualifications increases their likelihood of seeking work elsewhere. Here, rather than strengthening local retention, further training serves as a pathway to external opportunities [33,38,39]. This phenomenon reveals a low level of commitment to current workplaces and suggests that retention in Turkana may be temporary and conditional.

National ambitions for increased labour mobility further shape these dynamics. The Kenyan government’s goal of sending three million citizens abroad to work within the next three years to boost the national economy frames outmigration as both accessible and aspirational [40]. Many nurses in our study also portray outmigration as a pathway to economic and personal advancement that appeals to those feeling limited by local opportunities. However, if outmigration becomes more accessible, it may exacerbate existing strain on healthcare systems. In this context, the current impression of workforce stability in Turkana is fragile, and shaped by constrained mobility, emerging aspirations to leave and the existence of ghost workers that obscure true staffing pictures.

### Retention: global and local dynamics

The dynamics in Turkana mirror global trends in outmigration and their long-term impacts on local health systems. While outmigration provides immediate economic benefits for individuals, their families, and their country through remittances, these benefits can overshadow long-term national health system challenges such as workforce shortages and weakened service delivery [41]. Previous research shows that outmigration directly impacts workforce retention, as the migration of skilled healthcare workers undermines efforts to maintain a stable and adequately staffed system [42]. Kenya falls short of the WHO recommended minimum of 4.45 health workers per 1,000 people, with rural areas experiencing even higher shortages of health workers [43,44]. This gap is further exacerbated by the annual migration of over 4,000 healthcare workers [45].

The UK–Kenya Bilateral Labour Agreement (BLA) exemplifies the interplay of global push and pull factors affecting healthcare worker migration. Destination countries such as the UK, with ageing populations, increasing healthcare demands, and insufficient domestic training pipelines, have prioritised international recruitment as a key strategy for maintaining their healthcare systems [43]. Conversely, systemic challenges in source countries, like those we see in Kenya, push health workers to seek better opportunities abroad [43]. This dynamic illuminates a stark global imbalance: destination countries reap the benefits of workforce mobility and brain gain, while source nations face workforce shortages, systemic inequities, and brain drain [46].

This global healthcare worker mobility sharply contrasts the immobility observed in regions like Turkana, where structural barriers and constrained opportunities create seemingly stable retention rates. This juxtaposition raises important questions about what retention means in practice, and whose interests are ultimately served by current bilateral labour agreements. Although immobility may temporarily stabilise the workforce in places like Turkana, this is not sustainable. Conversely, though increased global mobility may address the needs of destination countries, it risks further undermining source nations’ already fragile health systems. Bilateral agreements therefore require closer evaluation, as they may reinforce rather than reduce existing global disparities.

To address the increasing workforce shortage, Kenya’s government has implemented targeted educational measures to expand the supply of licensed and active health professionals [47]. Between 2006 and 2015, Kenya’s health workforce increased by 68.5% [48], demonstrating the potential of education initiatives to strengthen the health system. However, existing evidence show that newly trained professionals often face unemployment or temporary employment due to resource constraints and the lack of specialised service infrastructure and may resort to volunteering unless they can secure general nursing roles or temporary locum positions [49,50]. This pattern is also reflected in our findings and a disconnect between workforce education and labour market absorption, underscoring the limitations of education as a standalone solution.

Since devolution, county governments have assumed responsibility for recruitment and health budget allocations [50]. Absorption is therefore highly dependent on local political priorities and fiscal capacity, which contributes to county disparities [50]. Against this backdrop, there is a growing global emphasis on workforce education as a tool for health system strengthening [24,51] and its potential to improve healthcare access and quality in rural areas [52]. However, the effectiveness of such investments depends on whether local systems can meaningfully absorb and retain trained health workers. If resources allocated to strengthening local health systems instead feed global demands through outmigration, educational initiatives risk exacerbating inequities rather than addressing them. Our findings in Turkana illustrate this tension; many female nurses described pursuing further qualifications as a potential pathway out of the county, and that, for those who remain, specialised training often goes unused due to lack of available positions. In such contexts, expanding education without addressing systemic constraints does little to improve retention or workforce stability, and risks inadvertently contributing to mobility instead.

### Implications

This study reveals a need to reframe how retention is approached and conceptualised in policy, research, and practice. It is necessary to consider the interplay between local and global migration flows, and how lived experiences, including individuals’ aspirations to leave, are necessary to acknowledge when interpreting numerical retention data. Local policy strategies should address the paradox of high unemployment alongside persistent staffing shortages and ensure that the growing number of trained nurses can be effectively employed within Turkana’s health system. Transparent, merit-based systems that lessen reliance on personal networks, for example creating opportunities for specialised nurses to utilise their skills locally, can reduce inequitable career advancement and empower nurses with limited connections. Enhancing coordination between county governments and facilitating easier transfers could also improve mobility, leading to increased family reunification. Furthermore, gender-sensitive policies that support mentorship programmes, paid leave, lactation breaks, and childcare services have proven to be essential for reducing gender-specific barriers to career progression and increasing female nurse retention. However, these strategies lack consistent nation-wide implementation.

While our study sheds new light on the concept of retention, further research is needed to develop a deeper understanding and inform evidence-based strategies that meaningfully engage with nurses’ realities.

## Limitations

This review possesses several limitations. Although participants demonstrated strong English proficiency, potential language barriers may have arisen. With more resources, it could have enriched the findings to conduct research in the nurses’ native language. Recruiting participants through community gatekeepers who were nurses in leadership positions may have shaped participation decisions, as hierarchical relationships could have created perceived pressure to participate. The fact that some nurses declined to participate reassured us that decisions were voluntary; however, alternate recruitment strategies could further minimise this risk. Finally, a longer fieldwork period may allow for both a broader and deeper understanding of the retention of female nurses in Turkana.

## Conclusion

This qualitative study demonstrates how retention of female nurses in Turkana is driven by a complex interplay of local structural barriers and global migration dynamics. Exploring the perspectives of those retained reveals a discrepancy between the numerical workforce data and the nurses lived experience. Rather than an indicator of job satisfaction, perceived workforce stability can be largely attributed to immobility caused by limited alternatives and systematic constraints. This immobility masks underlying dissatisfaction and leaves the healthcare system in Turkana vulnerable to outmigration. The existence of high unemployment alongside persistent staffing shortage puts a further strain on the health system.

The findings enrich the existing literature and challenge general assumptions about what influences retention in an arid, rural East African setting. They underscore the need for targeted policies to improve retention and rural career pathways for female nurses by addressing gendered and structural barriers to career advancement and global migration dynamics. Promoting the retention and advancement of female health professionals is essential for ensuring a more equitable and effective health workforce, and to improve health outcomes for the communities they serve.

## Data Availability

The participants of this study did not give written consent for their data to be shared publicly, so due to the sensitive nature of the research supporting data is not available.
